# Preventive and Therapeutic Effect of Metformin in Head and Neck Cancer: A Concise Review

**DOI:** 10.3390/jcm12196195

**Published:** 2023-09-25

**Authors:** Cyril Bouland, Xavier Vanden Eynden, Martin Lalmand, Thibaut Buset, Antoine Yanni, Rokneddine Javadian, Alexandra Rodriguez, Isabelle Loeb, Jérôme R. Lechien, Fabrice Journe, Sven Saussez, Didier Dequanter

**Affiliations:** 1 Department of Stomatology—Maxillofacial Surgery, CHU-Saint-Pierre, Université Libre de Bruxelles (ULB), 1000 Brussels, Belgiummartin.lalmand@stpierre-bru.be (M.L.);; 2 Department of Otorhinolaryngology—Head and Neck Surgery, CHU-Saint-Pierre, Université Libre de Bruxelles (ULB), 1000 Brussels, Belgiumsven.saussez@umons.ac.be (S.S.); 3 Laboratory of Human Anatomy and Experimental Oncology, Faculty of Medicine and Pharmacy, Research Institute for Health Sciences and Technology, University of Mons (UMONS), 7000 Mons, Belgium; 4 Laboratory of Oncology and Experimental Surgery, Jules Bordet Institute, Université Libre de Bruxelles (ULB), 1070 Brussels, Belgium

**Keywords:** metformin, head and neck cancer, systematic review, treatment, prevention

## Abstract

Background: Head and neck cancer (HNC) is a complex affection. Nowadays, conventional treatments are associated with many side effects, reducing the patient’s quality of life. Recent studies suggest that metformin, a first-line treatment for diabetes, could decrease cancer incidence and improve cancer-related survival rates. Methods: This systematic review summarizes important data from studies evaluating metformin’s contribution to preventing and treating HNC. Results: The results suggest a protective effect of metformin in HNC. However, no consensus has been found on its therapeutic effects. Metformin seems to confer an improved cancer-related survival rate in a diabetic population, but compared to a non-diabetic population, the review could not identify any advantages. Nevertheless, no studies presented a negative impact. Conclusion: In conclusion, the results of this systematic review suggest that HNC patients may benefit from metformin. Indeed, it would reduce the HNC incidence. However, more studies are required to evaluate the effect on cancer-related survival rates.

## 1. Introduction

Head and neck cancers (HNC) are the sixth most prevalent cancers in the world. Over 90% of the HNC are squamous cell carcinoma (SCC). Head and neck squamous cell carcinomas (HNSCC) are responsible for 600,000 cases and 380,000 deaths worldwide [1,2,3,4]. HNC arise from the epithelial linings of the oral cavity, pharynx, larynx, nasal cavity, or paranasal sinuses [4]. The main risk factors are tobacco and alcohol habits and coinfection with the high-risk human papillomavirus (HPV) 16, 18, 31, 33, and 45 [5]. HNC are a significant public health concern [6]. The HNC mortality depends on several factors: the disease site, staging, perineural invasion, extracapsular spread, and HPV status. The overall survival (OS) of advanced HNC patients remains low, even though new prognostic biomarkers have been highlighted or new therapeutics and refined risk stratification have been initiated [4,7]. Nowadays, 40 to 50% of HNC patients die in the five first years [4].

Many studies have demonstrated a relationship between diabetes and HNC; however, the mechanisms of action are not well understood [8]. Hyperglycaemia, hyperinsulinemia, insulin resistance, chronic inflammation, and/or microvascular disease related to diabetes could affect the development of a potential metastasis, and/or prognosis of HNC. Furthermore, it could be negatively correlated with tumour prognosis [8]. Indeed, increased glucose uptake and enhanced glycolysis are HNSCC’s distinctive hallmarks. The metabolic pathways could be therapeutic targets for HNC patients [9]. Metformin, the first-line treatment for type II diabetes, is an oral anti-hyperglycaemic biguanide, which inhibits mitochondrial complex I and oxidative phosphorylation [10]. Diabetic patients treated with metformin have a 40% reduced risk of cancer compared to diabetic patients not treated with metformin [10]. Metformin has already displayed a protective effect on several types of cancer such as colorectal cancer, hepatocellular carcinoma and even pancreatic cancer, decreasing both mortality and incidence [11,12,13].

In 2015, Rêgo et al., performed a systematic review evaluating the contribution of metformin in HNSCC treatment. The authors highlighted decreased locoregional recurrence and metastasis rates and improved OS and recurrence-free survival (RFS) rates. Furthermore, the concept of using metformin as a chemopreventive agent to control head and neck carcinogenesis was raised [2]. Several studies highlighted that cancer patients with type II diabetes presented a decreased mortality after anti-glycaemic regimen treatment [14]. Metformin suppresses tumour cell proliferation and leads to apoptosis, both in vitro and in vivo, in HNSCC. It decreases colony formation through the interruption of the cell cycle. Preclinical studies have demonstrated that metformin can prevent premalignant oral lesions’ conversion to SCC [9]. This review aims to evaluate the contribution of metformin to the prevention and treatment of HNC.

## 2. Materials and Methods

The studies were considered for the current systematic review based on the population, intervention, comparison, and outcome (PICO) framework [15].

### 2.1. Type of Studies

Prospective or retrospective clinical trials published in peer-reviewed journals were selected in this review. Studies were included if they explored the contribution of metformin in HNC. HNC could arise from either the oral cavity, pharynx, larynx, nasal cavity, or paranasal sinus epithelial linings.

### 2.2. Participants and Inclusion/Exclusion Criteria

Papers were included in the analysis if they specifically reported the contribution of metformin in the prevention and treatment of HNC. The contribution should consider either the incidence or the prognosis. Papers treating another antidiabetic drug or another cancer location other than head and neck were excluded. Only studies written in English were included.

### 2.3. Outcomes

The first outcome of the study was either the impact of metformin on the incidence and/or prognostic data (i.e., overall survival (OS) and recurrence-free survival (RFS)) in HNC patients. The first paragraph will exclusively include the articles covering the incidence. The second one will encompass the prognosis.

### 2.4. Intervention and Comparison

In the case of the study of the prognostic value of HNC, the authors were required to have treated their patients with classical surgical or conservative treatments.

### 2.5. Search Strategy

Two independent authors (CB and XVDE) searched in PubMed, Cochrane, and Scopus to identify articles published from January 1990 until June 2023 regarding the contribution of metformin in the prevention and treatment of HNC. Clinical studies were screened if they had a database, abstracts, available full texts, or titles that referred to these conditions. The following keywords were used: HNC, head and neck cancer, metformin, diabetes, prognosis, incidence; overall survival, and recurrence-free survival. After a critical analysis of the publication content, the final article selection was determined by CB and XVDE. In case of a disagreement, a third author (DD) was invited for the final decision. The review was conducted according to the PRISMA checklist for reviews and meta-analysis. Institutional review board approval was not required.

### 2.6. Epidemiological Characteristics and Outcomes

An analysis of the population and their characteristics (tobacco habits, alcohol habits, HPV/EBV status, age, sex and comorbidities) was performed, including the locations of tumour(s), the histology, the type of treatment, the follow-up duration, the results and the outcome of the included studies.

## 3. Results

**Figure 1:**
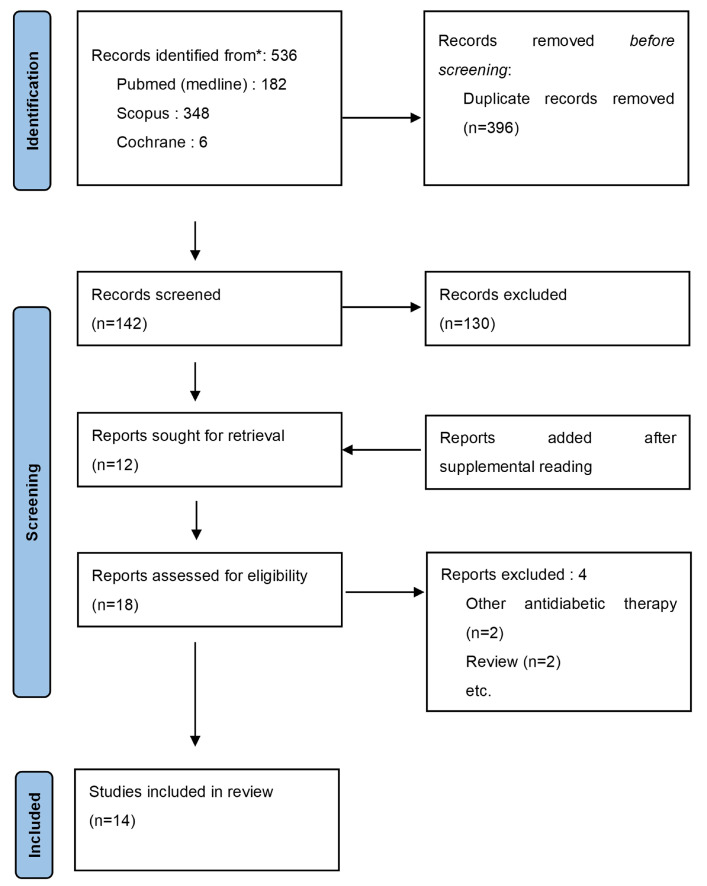
Selection process according to PRISMA guidelines.

**Figure 2:**
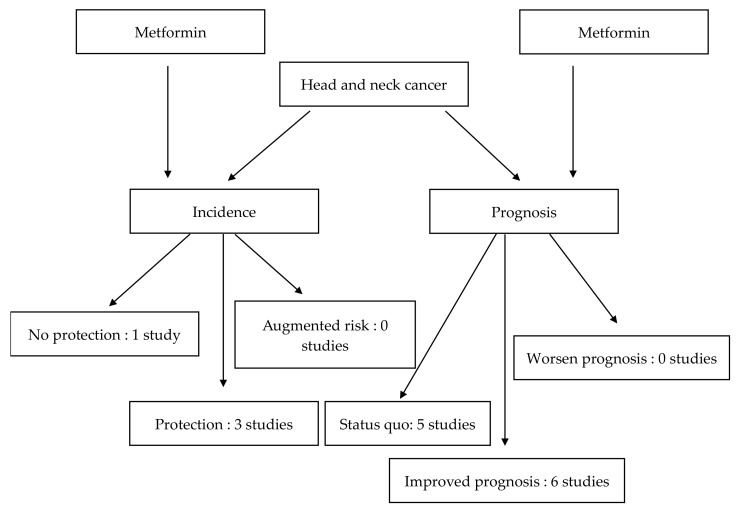
Flowchart of the results.

The search using the various keywords produced 536 results from the three databases. After the removal of duplicates, 142 articles were retained. After reading the abstract and title, 12 articles met the inclusion criteria. Six additional studies were included after browsing the literature. After reading the full text, four articles were excluded, two papers used other therapies and the other two articles were reviews. The final selection consisted of 14 articles (selection process: Figure 1). Four papers studied the protective effect of metformin against HNC [3,16,17,18] (Table 1 and Table 2). Eleven studies evaluated the impact of metformin in HNC treatment [3,4,14,19,20,21,22,23,24,25,26] (Table 3 and Table 4). One of the included articles studied both the incidence and prognosis [3]. Two of the included studies also assessed the toxicity of metformin [21,23]. The flowchart of the results is displayed in Figure 2.

### 3.1. Metformin Impact on HNC Incidence

Four studies analysed the HNC appearance and the protective effect of metformin [3,16,17,18] (Table 3). The first one did not observe a beneficial effect on HNC incidence [16]. However, the three others highlighted a protective effect [3,17,18]. The four studies are presented in two separate tables: Table 1: Characteristics of the population studied; and Table 2: Cancer characteristics and results of the clinical studies.

Becker et al., studied the impact of several antidiabetic drugs on the incidence of HNC. Unfortunately, neither metformin (1–29 prescriptions: adjusted OR 0.87, 95% CI: 0.61–1.24 and ≥30 prescriptions adjusted OR 0.80, 95% CI: 0.53–1.22), nor sulphonylureas (adjusted OR 0.87, 95% CI: 0.59–1.30), or any insulin use (adjusted OR 0.92, 95% CI 0.63–1.35) reduced the incidence of HNC during the long-term follow-up [16]. In contrast, the three following studies highlighted a protective effect. Yen et al., followed a newly diagnosed diabetic population of 66,600 patients for 10 years. Half of the population was treated with metformin (MET cohort) and the other half was not (ctrl cohort). After 10 years, the incidence of head and neck cancer was 34% lower in the MET cohort than in the ctrl cohort (adjusted hazard ratio (HR) 0.66; 95% confidence interval (CI): 0.55–0.79). Furthermore, the risks for oropharyngeal cancer (adjusted HR: 0.66; 95% CI: 0.17–0.74) and nasopharyngeal carcinoma (NPC; adjusted HR: 0.50; 95% CI: 0.31–0.80) were also significantly lower in the MET cohort than in the ctrl cohort [3]. Tseng observed that diabetic patients treated with metformin had a significantly reduced risk of developing oral cancer than diabetic patients treated without metformin, especially when the cumulative treatment time was more than 21.5 months [17]. Tseng highlighted similar results for nasopharynx cancer, especially after 24 months of cumulative metformin treatment [18].

### 3.2. Metformin Impact on HNC Prognosis

Eleven studies analysed the impact of metformin on HNC prognosis [3,4,14,19,20,21,22,23,24,25,26]. Six studies noticed a significant improvement in the HNC prognosis [4,14,19,20,21,22]. The other five studies did not highlight any prognosis improvement [3,23,24,25,26]. However, no study observed a prognosis worsening. The ten studies are presented in two separate tables: Table 3: Characteristics of the population studied; and Table 4: Cancer characteristics and results of the clinical studies.

#### 3.2.1. Prognosis: Improvement

In 2014, Sandulache et al., highlighted that a diabetic population treated with metformin that is suffering from a laryngeal squamous cell carcinoma, had a significantly greater OS compared to a diabetic population treated without metformin (OR, 3; 95% CI, 1.04–8.4; *p* = 0.04) and a non-significantly improved OS compared to the non-diabetic group ((OR), 2.23; (CI), 0.89–5.62; *p* = 0.09) [19]. Diabetic met+ presented a non-significantly improved DFS compared to both diabetic met− and non-diabetic groups (respectively, OR: 1.99; CI 0.82–4.83; *p* = 0.13 and OR: 1,77; CI: 0.85–3.68; *p* = 0.13). In 2019, Tsou et al., observed a non-significant OS and DFS (respectively, *p* = 0.67 and *p* = 0.68) differences between the diabetic and the non-diabetic groups, suffering from hypopharyngeal cancer, after a four-year follow-up [14]. However, significantly higher OS (*p* < 0.001) and DFS (*p* < 0.001) were highlighted in diabetic met+ patients compared to diabetic met– patients (respectively, 55.1% and 44.89%, compared to 27.90% and 60.46%). Multivariate analyses demonstrated that diabetic met+ patients suffering from advanced hypopharyngeal cell carcinoma, showed both improved OS (*p* < 0.01) and DFS (*p* < 0.01), compared to diabetic met− patients. Spratt et al., evaluated the impact of diabetes and metformin use in a population of oropharyngeal cancer patients treated with radiotherapy [20]. The authors highlighted no significant differences between non-diabetic patients, and diabetic patients treated with and without metformin in terms of local failure-free survival (LFFS), and regional failure-free survival (RFFS) after a five-year follow-up. However, the diabetic met− patients’ group had a significantly higher (*p* < 0.011) rate of distant metastases-free survival than nondiabetic patients. No differences were observed between the diabetic patients treated with metformin and nondiabetic patients. Multivariate analyses confirmed the results. Moreover, the 5-year actuarial rates of OS of diabetic met− patients was significantly worse than nondiabetic patients (*p* = 0.048). According to Alcusky et al., metformin has a protective effect but only during the first two years following the HNC diagnosis [4]. Indeed, the authors observed that all-cause mortality rate among diabetic met+ patients was 0.81 (95% CI: 0.61–1.09; *p* = 0.002) times lower compared to diabetic met− patients during the first two-years post-diagnosis. Notwithstanding, the all-cause mortality rate appeared to be higher (HR: 1.20, 95% CI: 0.94–1.53) among the diabetic patients treated with metformin after that period of two years. Furthermore, this association seemed to be more robust in the subgroup of patients younger than 60 years old (HR for 0–2 years post-diagnosis: 0.22, 95% CI 0.09–0.56; HR for ≥2 years post-diagnosis: 0.56, 95% CI 0.26–1.22). Indeed, this association was attenuated in patients over 60 years old (HR for 0–2 years post-diagnosis: 0.98, 95% CI 0.72–1.34; HR for the period ≥ 2 years post-diagnosis: 1.30, 95% CI 1.01–1.69). Treatment with metformin before the diagnosis of HNC was not associated with better survival (HR: 1.06, 95% CI 0.81–1.39). Interestingly, Gulatti et al., also observed an improved OS and RFS, after two years of follow-up, but in a non-diabetic population treated with the combination of radiotherapy, chemotherapy and metformin, suffering from a locally advanced head and neck squamous cell cancer (LAHNSCC), compared with the historical control rates (respectively, 90% and 85% compared to 80% and 65%) [21]. Stokes et al., highlighted that HNC diabetic met+ patients had a significantly improved OS (*p* < 0.01) and CSS (*p* < 0.01) compared to both non-diabetic patients and diabetic met− patients (respectively, 73.4%, 65.6%, 57.7% and 88.8%, 73.7%, 66.1%) after a two-year follow-up [22]. Multivariate analyses did not confirm univariate analyses. Indeed, non-diabetic patients and diabetic met− patients experienced significantly worse CSS as compared to diabetic met+ patients (respectively, HR 2.33, 95% CI 1.16–4.65, *p* = 0.02 and HR 3.03, 95% CI 1.49–6.16, *p* < 0.01) but neither the non-metformin group nor the non-diabetic group experienced significantly different OS than the diabetic patients under metformin group (non-diabetic group: HR 1.13, 95% CI 0.78–1.65, *p* = 0.53; non-metformin group: HR 1.36, 95% CI 0.92–2.00, *p* = 0.12).

#### 3.2.2. Prognosis: Status Quo

In 2015, Yen et al., did not demonstrate any significant difference in OS between diabetic patients treated with or without metformin who subsequently developed HNC [3]. In 2017, Chang et al., highlighted that metformin administration improves neither the OS nor the recurrence-free survival (RFS) of HNC patients [23]. Indeed, no statistical difference was observed in the one-year (71.8% vs. 76.1% *p* = 0.815) and the two-year RFS (69.2% vs. 60.2% *p* = 0.367) or the one-year (83.6% vs. 82.1% *p* = 0.570) and two-year OS (71.8% vs. 64.3% *p* = 0.305) between the metformin and the non-metformin diabetic groups. In 2018, Quimby et al., observed that metformin intake for at least one year, at the time of HNSCC diagnosis, in a diabetic population, does not improve OS (*p* = 0.9182) nor disease-specific survival (DSS) (*p* = 0.9918) of HNSCC patients compared to a non-diabetic population [24]. These results were confirmed by multivariate analyses (OS: HR 1.123, *p* = 0.338; DSS: HR 1.048, *p* =0.0792). In 2019, Lee et al., observed no significant differences between the metformin and the non-metformin users in an HNSCC diabetic population, not only in terms of OS (*p* = 0.83), DSS (*p* = 0.58), RFS (*p* = 0.88) but also in local (*p* = 0.22) or regional (*p* = 0.98) control or distant metastasis (*p* = 0.7) at the five-year follow-up [25]. Furthermore, no significant differences were highlighted, in any of the subsites: oral cavity, oropharynx, larynx. Kwon et al., observed that the diabetic population suffering from HNC not treated with metformin, had a significantly lower OS (*p* = 0.017) compared to the metformin-treated diabetic population and non-diabetic population [26]. The diabetic population not treated with metformin also presented a lower cancer-specific index, even if the *p*-value was at the limit of significance (*p* = 0.054). No statistical difference in CSS was observed between diabetic patients treated with or without metformin and non-diabetic patients (*p* = 0.2). Furthermore, the authors evaluated cause-specific survival. Higher cumulative incidences of index HNC-specific death were observed in diabetic patients not treated with metformin compared with nondiabetic patients (HR: 1.95; 95% CI: 1.03–3.72; *p* = 0.041). Diabetic patients treated with metformin had better survival outcomes related to cancer-specific death compared to diabetic patients treated without metformin (HR: 0.45; 95% CI: 0.20–0.99; *p* = 0.047). However, outcomes were worse than those of non-diabetic patients (HR: 0.88; 95% CI: 0.42–1,83; *p* = 0.072). However, no significantly improved OS nor CSS were confirmed by the multivariate analyses.

### 3.3. Toxicity

Metformin posology was described in two studies [21,23]. The first one observed no significant difference in prognosis with patients treated with 500–2000 mg (median: 1500 mg) [23]. Interestingly, the 39 patients treated with metformin supported a significantly lower cumulative dose of cisplatin (161.0 ± 8.8 mg/m^2^ (Met+) vs. 197.1 ± 89.8 mg/m^2^ (Met−); *p* = 0.038) and radiotherapy (65.6 ± 11.5 Gy vs. 69.1+/−9.1 Gy; *p* = 0.095) or concomitant CRT compared to the 213 patients (only four of whom were diabetic) not treated with metformin. Patients treated with metformin lost significantly more weight during simultaneous CRT (25.9 6 5.0% vs. 23.8 6 6.6; *p* = 0.027). Therefore, nutritional support is needed more often in these groups (74.4% vs. 58.7%; *p* = 0.060). Last but not least, patients presented grade ≥ 3 toxicities: nausea, vomiting, and haematological toxicities. However, these toxicities were mainly related to standard-of-care treatment rather than metformin. The other study was a phase I study [21]. Eighteen non-diabetic patients with a locally advanced HNSCC received a daily dose of 2000 mg, 2550 mg, and 3000 mg in split doses in addition to CRT for seven to 14 days. No death was reported during the trial. However, only ten patients completed the trial due to signs of gastrointestinal tract toxicity. A daily dose of 2550 mg of metformin, in association with CRT, was found to be the highest tolerable intake.

## 4. Discussion

The current study investigated the effect of metformin on HNC incidence and prognosis. We have included 14 clinical studies. One study was included in both parts. Four of them analysed the protective effect of metformin on HNC incidence, eleven studied the protective effect on its prognosis in terms of indicators of cancer-related survival rate and one study evaluated both effects on HNC.

Four studies [3,16,17,18] evaluated the risk of developing HNC in a population treated with metformin. Metformin did not increase the HNC incidence. Three studies observed a significantly decreased incidence of HNC [3,17,18] and the last one [16], did not show any significant effect of metformin. In the four studies, all the patients treated with metformin suffered from diabetes. The two studies published by Tseng highlighted a significantly decreased incidence of oral cancer and NPC [17,18]. The four studies obtained their data from national databases. However, the number of patients treated with metformin in the study group in the article by Becker et al., was small compared to the other three studies. Indeed, in the study by Becker and colleagues, the 2874 cases of HNC were matched with 17,244 controls from the Clinical Practice Research Datalink (CPRD). However, only 214 and 1273 patients, presented diabetes in, respectively, the HNC and control group. Only 112 patients were treated with metformin in the HNC group and 802 in the control group [16]. The three other studies included, respectively, 288,198 [17] 15,486 [18] and 33,300 [3] metformin users. The difference in the sample size could explain the discrepancy in the results. The duration of the metformin intake may, or may not, play a significant role. Tseng observed a significantly decreased incidence of oral cancer and NPC, especially after, respectively, 21,5- and 26,03-months intake [17,18]. On the opposite side, Becker et al., did not highlight any significantly decreased incidence in their study even though 90% of the patients on long-term metformin (≥30 prescriptions) developed diabetes more than four years before [16].

Cancer-related survival rates are displayed in eleven studies with varying degrees of success [3,4,14,19,20,21,22,23,24,25,26]. The results of the included studies were given with different peculiarities. The articles deliver results in terms of OS, DFS, RFS and DSS, RFFS, and LFFS, making the comparisons difficult. We eventually compared cancer-related survival rates. Six studies [4,14,19,20,21,22] observed significantly improved cancer-related survival rates and five [3,23,24,25,26] were non-significant. The last study also observed an improved OS and RFS compared to historical control rates [21]. No study highlighted decreased cancer-related survival rates in the studied cohorts. Interestingly, metformin seems to protect against metastases [20]. Ogunsakin et al., highlighted similar results in a letter to the editors published in 2018. The authors investigate the potential therapeutic benefit of metformin therapy in diabetic patients with SCC of the larynx and oropharynx after a five-year follow-up [27]. They observed that not only was the OS significantly higher in the metformin-treated group (*p* < 0.038), but also a significantly reduced risk of metastasis (*p* < 0.001) was demonstrated. Nearly 73% (72.7%) of the metformin-treated patients survived after five years compared to the other group (34.7%). Diabetic patients treated with metformin developed metastases in only 18.1% of the cases compared to diabetic patients treated without metformin (82.6%).

We observed heterogeneity between the results of the different studies. However, metformin seems to confer an advantage in a diabetic population [4,14,19,22]. Nevertheless, this review could not assess if metformin intake provides any advantage compared to a non-diabetic population. Interestingly, Alcusky et al., pointed out a notion of time: two years. Indeed, the authors observed that in the first two years after the HNC diagnosis, the all-cause mortality rate among metformin-exposed patients was 0.81 times the rate among unexposed patients [4]. The other studies did not observe any limitation in time for the contribution of metformin in the HNC treatment [14,19,22].

Metformin demonstrated its activity against multiple oncogenic pathways [10,28]. Cancer cell proliferation could be suppressed at several levels through either the inhibition of its metabolism or the activation of adenosine monophosphate-activated protein kinase (AMPK). Indeed, AMPK activation inhibits the protein mammalian target of rapamycin (mTOR). mTOR controls cell growth through mRNA translation and ribosome genesis. Hence, direct inhibition of AMPK prevents mTOR activation and thus, suppresses downstream cell proliferation and carcinogenesis [21,23]. Metformin has been suggested to be a direct tumour growth inhibitor by downstream suppression of signalling through mTOR [21]. Furthermore, metformin induces cell cycle arrest in the G0/G1 phase and apoptosis of cancer cells, thereby giving insight into possible mechanisms of metformin-mediated anticancer effects [2]. Metformin lowers the mitogenic activity of hyperinsulinemia through a reduction in systemic levels of insulin and insulin-like growth factor 1 (IGF-1) and displays an anti-neoplastic effect [25]. Additionally, metformin decreases oxidative stress, causing less DNA damage and mutagenesis [29]. Various studies observed that metformin is radiosensitising in the case of colorectal and oesophageal cancers by causing G2/M phase arrest, in pancreatic cancer by inhibiting DNA repair, abrogating the G2 phase checkpoint, in oesophageal cancer by activating ATM and AMPK, and in HCC by abrogating the G2/M phase arrest [14].

The literature presents limited data on metformin toxicities and treatment tolerance during HNC treatment. Chang et al., observed a decreased tolerance and increased toxicity of concurrent CRT in HNC patients [23]. These results are opposed to those of Kuo et al., who highlighted a protective effect of metformin against the cytotoxic effects of cisplatin in vitro [30]. Additional precautions for potential adverse events should be implemented when prescribed accordingly. Nevertheless, offering supportive care and nutritional intervention is critical during the therapeutic course [23]. Indeed, a declined quality of life or physical condition, but also tolerability or toxicity of the treatment is associated with a poor nutritional status before and during treatment. The use of a prophylactic percutaneous endoscopic gastrostomy could counteract this weight loss and poor recovery [31]. Notwithstanding, metformin has generated extensive interest after preclinical studies’ results. Metformin seems to improve the prognosis in different cancers such as lung, colorectal, prostate, breast, kidney or pancreatic cancers [16,29]. Currently, several clinical trials (Home-ClinicalTrials.gov) are progressing to evaluate both treatment and prevention: prevention against potentially malignant oral lesions (NCT03684707) [32] or premalignant lesions (NCT02581137) [33] (NCT05237960) [34], treatment in association with other treatments such as surgery and doxycycline (NCT03076281) [35], or surgery and durvalumab (NCT03618654) [36]. However, clinical evidence for supporting metformin’s contribution to survival benefits in patients with HNC is inconsistent. Thus, prospective comparative studies with a large size sample are needed to confirm the results of this review.

### Strengths and Weakness

Several limitations can be highlighted in this review. The populations studied are variable. Different risk factors influence HNC prevalence and prognosis such as tobacco, alcohol, betel nut chewing, HPV, and EBV infections. Unfortunately, these are not always described in the articles. Depending on the anatomopathology, the staging, and its location, the therapeutic protocol varies. Treatment relies principally on surgery, radiotherapy, and chemotherapy [8]. The diabetic status should deserve more attention. Studies do not explain whether patients have controlled diabetes or not [16]. Indeed, hyper- or hypoglycaemia has a direct impact on tumour growth. All but one of the studies included only diabetic patients in the metformin groups [21]. It would have been interesting to assess the contribution of metformin in a non-diabetic population. Metformin treatment, the first-line treatment for type II diabetes, itself also presents several limitations. Metformin cannot always be prescribed. Poor liver or renal function, old-age or even suffering from gastrointestinal side effects are contra-indications/exceptions to the recommendation of metformin. Furthermore, metformin should go along with regular physical activities, diet control, blood-sugar monitoring and of course medication compliance which are rarely followed thoroughly, by a significant proportion of diabetic patients. Consequently, this proportion of patients will be subject to long-term exposure to both hyperglycaemia and hyperinsulinemia, two risk factors fostering tumour growth. In consequence, low compliance creates bias [3].

## 5. Conclusions

In conclusion, this review highlighted metformin’s beneficial contribution to HNC. We have observed a positive association between metformin and HNC. Patients treated with metformin present a lower incidence of HNC. Metformin seems to confer improved cancer-related survival rates in a diabetic population compares to a non-diabetic population. Moreover, metformin seems to protect against metastases. The review cannot assess any advantages of metformin. However, more studies are needed to precisely evaluate the effect on cancer-related survival rates. Nevertheless, the use of metformin in HNC prevention or therapy should cautiously be monitored. The metformin anti-cancer activity should be well-defined via rigorous preclinical and observational investigations in both diabetic and non-diabetic populations before its implementation in the therapeutic arsenal of HNC. Both fundamental research to understand the underlying mechanism of metformin and further clinical and observational trials are mandatory to better understand metformin’s contribution to HNC treatment and prevention.

## Figures and Tables

**Table 1:** Characteristics of the population studied.

Study	Patients (N)	Tobacco Habits	Alcohol Habits	HPV/EBV Status	Age (Years)	Sex (M/F)	Comorbidities
Becker et al., 2014 [16]	Cases: 2874 HNC Diabetes: 214 HNC Diabetes Met−: 103 HNC Diabetes Met+: 111 1–29 prescriptions: 61 ≥30 prescriptions: 50 HNC Diabetes Ctrl: 17,244 Ctrl Diabetes: 1273 HNC HNC Diabetes Met−: 560 HNC Diabetes Met+: 713 1–29 prescriptions: 392 ≥30 prescriptions: 321	HNC: Non-smoker: 835 Current: 973 Past: 720 Unknown: 346 Ctrl: Non-smoker: 7583 Current: 3135 Past: 4704 Unknown: 1822	HNC: Never: 384 Current: 1993 Past: 59 Unknown: 438 Ctrl: Never: 2582 Current: 11,954 Past: 225 Unknown: 2483	/	HNC <40: 160 40–59: 1011 60–69: 771 70–79: 652 80–89: 280 Ctrl: <40: 900 40–59: 5944 60–69: 4601 70–79: 4008 80–89: 1791	HNC: M: 1834 F: 1040 Ctrl: M: 11,004 F: 6240	HNC CHF: 73 IHD: 312 Hypertension: 851 Stroke/TIA: 164 Dyslipidaemia: 300 Diabetes: 216 EBV: 10 Asbestosis: 12 Ctrl: CHF: 405 IHD: 1928 Hypertension: 5173 Stroke/TIA: 900 Dyslipidaemia: 1939 DM: 1343 EBV: 86 Asbestosis: 46
Tseng 2016 [17]	Original sample: Met+: 288,198 Oral cancer: 1273 Met−: 16,263 Oral cancer: 119 Matched sample: Met+: 16,263 Met−: 16,263	Original sample Met+: Smoker: 5915 Non-smoker: 282,283 Met−: smoker: 266 Non-smoker: 15,997 Matched sample Met+: smoker: 247 Non-smoker: 16,016 Met−: Smoker: 266 Non-smoker: 15,997	Original sample Met+: Abuser: 15,451 Non-abuser: 272,747 Met−: Abuser: 1037 Non-abuser: 15,226 Matched sample Met+: Abuser: 1059 Non-abuser: 15,204 Met−: Abuser: 1037 Non-abuser: 15,226	/	Original sample: Met+: 56.6 (+/−10.2) Met−: 59.1 (+/−10.4) Matched sample: Met+: 59.4 (+/−9.7) Met−: 59.1 (+/−10.2)	Original sample: Met+: M: 155,199 F: 132,999 Met−: M: 9332 F: 6931 Matched sample: Met+: M: 9437 F: 6826 Met−: M: 9332 F: 6931	Original sample Met+: Hypertension: 198,483 Dyslipidaemia: 197,488 COPD: 110,809 Diabetes-related complications: Nephropathy: 46,223 Eye disease: 41,653 Stroke: 54,814 IHD: 98,033 PAD: 45,915 Met−: Hypertension: 11,995 Dyslipidaemia: 9855 COPD: 6521 Diabetes-related complications: Nephropathy: 4139 Eye disease: 1529 Stroke: 4021 IHD: 6218 PAD: 2516 Matched sample Met+: Hypertension: 12,033 Dyslipidaemia: 9690 COPD: 6509 Diabetes-related complications: Nephropathy: 4123 Eye disease: 1341 Stroke: 3947 IHD: 6256 PAD: 2505 Met−: Hypertension: 11,995 Dyslipidaemia: 9855 COPD: 6521 Diabetes-related complications: Nephropathy: 4139 Eye disease: 1529 Stroke: 4021 IHD: 6218 PAD: 2516
Tseng 2018 [18]	Met+: 15,486 Met−: 15,486	/	/	/	Met+: <50: 1415 >50: 14,071 Met−: <50: 1654 >50: 13,832	Met+: M: 8898 F:6588 Met− M: 8953 F: 6533	Met+ Nephropathy: 4840 Liver disease: 3288 Oesophagus, stomach, duodenum diseases: 13,361 Met−: Nephropathy: 4910 Liver disease: 3357 Oesophagus, stomach, duodenum diseases: 13,340
Yen et al., 2014 [3] *	Diabetic Met + 33,300 Diabetic Met − 33,300	/	/	/	Met+: <40:4452 40–65: 18,321 >65: 10,527 Met−: <40:4452 40–65: 18,321 >65: 10,527	Met+: F: 16,287 M: 17,013 Met−: F: 16,287 M: 17,013	Met+: Coronary artery disease: 2507 Obesity: 65 CKD: 555 Hyperlipidaemia: 1388 Hypertension: 8663 Met−: Coronary artery disease: 2507 Obesity: 65 CKD: 555 Hyperlipidaemia: 1388 Hypertension: 8663

* Yen et al., 2014, evaluated both the effect on the prognosis and the prevention of HNC and is described in Table 1. Abbreviations: CHF: Congestive heart failure; CKD: Chronic kidney disease COPD: Chronic obstructive pulmonary disease; Ctrl: control; EBV: Epstein–Barr virus infection; F: Female; HNC: Head and neck cancer; IHD: Ischaemic heart disease; M: Male Met−: not treated with metformin; Met+: treated with metformin; PAD: Peripheral arterial disease; TIA: Transient ischaemic attack.

**Table 2:** Cancer characteristics and results of the clinical studies.

Study	Cancer Location	Histology (Cancer)	Treatment	Follow-Up	Results	Conclusion
Becker et al., 2014 [16]	HNC: 2874 Oral cavity: 1206 Pharynx: 570 Larynx: 680 Nasal cavity: 73 Unknown: 345	/	Documented in 84% of the HNC cases: Surgery Radiotherapy Chemotherapy	Mean: 10.6 (+/−4.7) years	No significantly decreased incidence of HNC in a metformin treated population regardless of the number of prescriptions.	Metformin is not associated with an altered risk of HNC.
Tseng 2016 [17]	Oral cavity	/	/	At least 6 months	Oral cancer Incidence is significantly lower in a Met+ population compared to a Met− population, especially when the cumulative duration is >21.5 months.	Metformin significantly reduces the risk of oral cancer.
Tseng 2018 [18]	Nasopharynx	/	/	Met+: 5 (0.5–6) years Met−: 5.4 (0.5–6) years	NPC incidence is significantly lower in a Met+ population compared to a Met− population, especially when the cumulative duration is >26.03 months.	Metformin significantly reduces the risk of NPC.
Yen et al., 2014 [3] *	Diabetic Met+: HNC: 195 Oral cavity: 118 Oropharynx: 10 Salivary glands: 3 Nasopharynx: 26 Hypopharynx: 19 Rhinosinus: 5 Larynx: 14 Diabetic Met−: HNC: 290 Oral cavity: 142 Oropharynx: 27 Salivary glands: 7 Nasopharynx: 52 Hypopharynx: 32 Rhinosinus: 10 Larynx: 20	/	/	/	HNC incidence is 0.64 times lower in Met+ patients compared to Met− patients. HNC incidence in Met+ 40–65 years and >65 years subgroups are significantly lower than in the Met− 40–65 years and >65 years subgroups. No significant difference in OS between patients with diabetes in the Met+ and Met− cohorts who subsequently developed HNC. The risks for oropharyngeal cancer and NPC are significantly lower in the Met+ cohort compared to the Met− cohort.	Metformin reduces the OS of HNC in diabetic patients.

* Yen et al., 2014 evaluated both the effect on the prognosis and the prevention of HNC which is described in the prevention part (A). Abbreviation: HNC: Head and neck cancer; Met+: Patients treated with metformin; Met−: Patients not treated with metformin; NPC: Nasopharyngeal cancer; OR: Overall survival; OS: overall survival.

**Table 3:** Characteristics of the population studied.

Study	Patients (N)	Tobacco Habits	Alcohol Habits	HPV/EBV Status	Age (Years)	Sex (M/F)	Comorbidities
Alcusky et al., 2019 [4]	HNC: 7872 Met+ (HNC diagnosis): 436 Other diabetes medications (HNC diagnosis): 456 Met+ (after HNC diagnosis): 708	/	/	/	Median: 68.1 (15.3–68.3)	M: 5954 F: 1918	/
Chang et al., 2017 [23]	HNC: 252 Diabetes: 43 Met+: 39 Met−: 4 No diabetes: 209	Met+: 31 Smokers 8 Non-smokers Met− (includes non-diabetic patients): 175 Smokers 38 Non-smokers	Met+: 27 Abusers 12 Non-abusers Met− (includes non-diabetic patients): 153 Abusers 60 Non-abusers	/	Met+: 56.1 (+/−12.2) Met− (includes non-diabetic patients): 52.3 (+/−10.9)	Met+: M: 35 F: 4 Met− (includes non-diabetic patients): M: 197 F: 16	/
Gulati et al., 2019 [21]	18	11 Smokers 7 Non-smokers	/	p16+: 13 p16−: 5	56 (46–65)	M: 15 F: 3	/
Kwon et al., 2015 [26]	HNSCC: 1151 Non-diabetic group: 973 Diabetic group: 178 Met+: 99 Met−: 79	/	/	/	61	M: 991 F: 160	/
Lee et al., 2019 [25]	HNSCC: 329 Met+: 195 Met−: 134	Met+ Never: 42 Current/Ex: 151 Missing: 2 Met−: Never: 32 Current/Ex: 101 Missing: 1	Met+: Never/Light: 121 Moderate/Heavy/Ex: 57 Missing: 17 Met−: Never/Light: 67 Moderate/Heavy/Ex: 54 Missing: 13	OPC group: Known p16 status: 67 p16+: 47 p16−: 20 Unknown p16 status: 50	Met+: 67.3 (+/−9.8) Met−: 67.6 (+/−9.7)	/	/
Quimby et al., 2018 [24]	HNSCC: 1231 Met+: 165 Met− l: 1066 Met−: No Met at least 1 year before and at least 1 year after cancer diagnosis	/	/	/	Met+: 74.55 (+/−6.09) 65–69: 38 70–74: 49 75–79: 44 80–84: 25 85–90: 6 >90: 3 Met−l: 74,51 (+/−6.35) 65–69: 280 70–74: 289 75–79: 257 80–84: 168 85–90: 52 >90: 20	Met+: M: 141 F: 24 Ctrl: M: 891 F: 175	/
Sandulache et al., 2014 [19]	Non-diabetic: 162 Diabetic: 43 Met+: 21 Met−: 22	Non-diabetic: 162 Smoker: 157 Non-smoker: 5 Diabetic Met+: 21 Smoker: 20 Non-smoker: 1 Diabetic Met−: 22 Smoker: 22 Non-smoker: 0	Non-diabetic: 162 Alcohol: 127 Non Alcohol: 35 Diabetic Met+: 21 Alcohol: 18 No alcohol: 3 Diabetic Met−: 22 Alcohol: 17 No Alcohol: 5	/	Non-diabetic: 63(Mean) Diabetic: 65 (Mean) Diabetic Met+: 64 (Mean) Diabetic Met−: 66 (Mean)	/	/
Stokes et al., 2018 [22]	Non-diabetic: 1144 Diabetic: 502 Diabetic Met+: 124 Diabetic Met−: 378	/	/	/	Non-diabetic: 66–69: 286 70–74: 325 ≥75: 533 Diabetic Met+: 66–69: 30 70–74: 33 ≥75: 61 Diabetic Met−: 66–69: 84 70–74: 97 ≥75: 197	/	Hypertension/Chronic kidney disease Non-diabetic: Yes: 801 No: 343 Diabetic Met+: Yes: >113 No: <11 Diabetic Met−: Yes: 334 No: 44 Hyperlipidaemia Non-diabetic: Yes: 693 No: 451 Diabetic Met+: Yes: 103 No: 21 Diabetic Met−: Yes: 300 No: 78
Tsou et al., 2019 [14]	Non-diabetic: 49 Diabetic: 92 Met+: 49 Met−: 43	Non-diabetic: 49 Smoker: 28 Non-smoker: 21 Diabetic Met+: 49 Smoker: 33 Non-smoker: 16 Diabetic Met−: 43 Smoker: 31 Non-smoker: 12	Non-diabetic: 49 Alcohol: 26 No Alcohol: 13 Diabetic Met+: 49 Alcohol: 28 No alcohol: 21 Diabetic Met−: 43 Alcohol: 16 No Alcohol: 27	/	Non-diabetic: 63.28 Diabetic Met+: 66.45 Diabetic Met−: 65.04	/	/
Spratt et al., 2016 [20]	Non-Diabetic: 1560 Diabetic: 184 Met+: 102 Met−: 82	1735	/	HPV+: 371 HPV−: 139 Unknown: 1235 P16+: 366 p16−: 19 Unknown: 1340	56 (25–91)	M: 1520 F: 225	/

Abbreviations: CAD: coronary artery disease; CHF: congestive heart failure; CKD: chronic kidney disease; EBV, Epstein–Barr virus infection; F: Female; HNC: Head and Neck Cancer; HNSCC: Head and neck squamous cell carcinoma; IHD: ischaemic heart disease; M: Male; Met−: not treated with metformin; Met+: treated with metformin; OPC: Oropharyngeal cancer; TIA, transient ischaemic attack.

**Table 4:** Cancer characteristics and results of the clinical studies.

Study	Location (Cancer)	Histology (Cancer)	Treatment	Follow-Up (Months)	Results	Conclusion
Alcusky et al., 2019 [4]	OPSCC: 873 Larynx: 3192 Undefined HNC: 3807	/	Surgery: 5528 (alone/combination) Radiotherapy: 3822 Chemotherapy: 2549	median follow-up: 35.2 (15.3–68.3) months	Metformin has a protective effect but only during the first two years following the HNC diagnosis. The all-cause mortality rate among Met+ patients is 0.8, especially in the patient sub-group 60 years and younger. Metformin exposure prior to the HNC diagnosis is not associated with a better survival.	Metformin is associated with a lower rate of all-cause mortality during the first two years after diagnosis. Age seems to modify the association between metformin and HNC survival.
Chang et al., 2017 [23]	HNC: 252 Met+: Oral cavity: 20 Oropharynx: 9 Hypopharynx: 10 Met−: Oral cavity: 81 Oropharynx: 71 Hypopharynx: 60	SCC	CRT	36 months	No significant difference of OS or RFS during the two-years follow-up between Met+ and Met− groups.	Metformin in HNC patients is not associated with an improved OS or RFS.
Gulati et al., 2019 [21]	Oropharynx: 12 Larynx: 6	SCC	CRT	median follow-up: 19 months	The treatment combining CRT and metformin improves OS and PFS compared to the historical OS and PFS rates. The most common grade ≥3 toxicities (diarrhoea (6%), nausea (11%), vomiting (11%), mucositis (6%), acute kidney injury (17%), anaemia (6%), and leukopenia (11%)), were mainly related to standard-of-care treatment rather than metformin.	First phase 1 trial combining metformin with CRT. OS and PFS rates were encouraging in this limited patient population and warrant further investigation in a phase 2 trial.
Kwon et al., 2015 [26]	HNC	SCC	Surgery Radiotherapy Chemotherapy	65.1 (12.1–154.5) months	Metformin use does not improve the OS nor CSS in HNC patients.	Metformin treatment did not improve survival of HNC patients.
Lee et al., 2019 [25]	Met+: Oral Cavity: 68 Oropharynx: 44 Hypopharynx: 8 Larynx: 75 Met−: Oral Cavity: 49 Oropharynx: 36 Hypopharynx: 5 Larynx: 44	SCC	Met+: Surgery: 43 Adjuvant Rad: 24 Adjuvant Chemo/Rad: 6 Primary Rad: 93 Primary Chemo/Rad: 29 Met−: Surgery: 30 Adjuvant Rad: 14 Adjuvant Chemo/Rad: 1 Primary Rad: 66 Primary Chemo/Rad: 23	Met+: 3,1 (+/− 2.1) years Met−: 3 (+/− 2.2) years	Metformin use was not significantly associated with improved OS, RFS and DSS at 5-years follow-up.	No association between metformin use and oncologic outcomes were observed.
Quimby et al., 2018 [24]	Met+: Nasopharynx: 12 Hypopharynx: 17 Glottic larynx: 105 Supraglottic larynx: 31 Met−: Nasopharynx: 55 Hypopharynx: 152 Glottic larynx: 656 Supraglottic larynx: 203	SCC	Met+: Surgery ± RT/CRT: 31 RT ± Surgery: 116 CRT ± Surgery: 18 Met−: Surgery ± RT/CRT: 266 RT ± Surgery: 646 CRT ± Surgery: 154	/	Metformin does not improve OS nor DSS in HNSCC patients.	Metformin does not give a survival advantage to HNSCC patients.
Sandulache et al., 2014 [19]	All: Glottic larynx: 120 Supraglottic larynx: 85 Non-diabetic: Glottic larynx: 88 Supraglottic larynx: 74 Diabetic Glottic larynx: 32 Supraglottic larynx: 11 Diabetic Met+: Glottic larynx: 17 Supraglottic larynx: 4 Diabetic Met−: Glottic larynx: 15 Supraglottic larynx: 7	SCC	/	/	Metformin users demonstrated a significantly improved OS compared to diabetic patients treated without metformin and a non-significant improved OS compared to non-diabetic patients.	Diabetic patients taking metformin during treatment for Laryngeal SCC exhibited improved clinical outcome compared to diabetic patients treated without metformin.
Stokes et al., 2018 [22]	Oral Cavity: 835 Non-diabetic: 583 Met+ diabetic: 72 Met− diabetic: 180 Hypopharynx: 585 Non-diabetic: 397 Met+ diabetic: 35 Met− diabetic: 153 Other: 226 Non-diabetic: 164 Met+ diabetic: 17 Met− diabetic: 45		Surgery Radiotherapy Chemotherapy	/	Non-diabetic patients and diabetic patients treated without Met experience significantly worse CSS compared to diabetic patients treated with Met. However, No OS differences are observed in the three groups.	Diabetic HNC patients treated with metformin experience improved CSS.
Tsou et al., 2019 [14]	Hypopharynx	/	CRT	48 months	Advanced hypopharyngeal SCC Met+ diabetic cohort exhibit significantly improved OS and DFS compared to Met− diabetic cohort.	Advanced hypopharyngeal SCC diabetic patients treated with metformin exhibit improved OS and better DFS.
Spratt et al., 2016 [20]	Oropharynx: 1745 (total) Tonsil: 805 Non-diabetic: Met+ diabetic: Met− diabetic: Base of the tongue: 845 Non-diabetic: Met+ diabetic: Met− diabetic: Soft palate: 22 Non-diabetic: Met+ diabetic: Met− diabetic: Pharyngeal wall 61 Non-diabetic: Met+ diabetic: Met− diabetic: Others: 16 Non-diabetic: Met+ diabetic: Met− diabetic:	SCC	Radiotherapy Chemotherapy	51.6 (5-year actuarial rates)	Diabetic patients treated with Met+ present a 5-year DMFS (90.1%) and OS (89.6%), similar to non-diabetic patients. Multivariate analysis (reference diabetic treated without metformin) demonstrated improved DMFS for non-diabetic patients and a trend toward improved DMFS with met+ users. LFFS and RFFS are high in all groups and are not significantly different by diabetic status or metformin use.	Diabetic patients not using metformin independently have significantly higher rates of distant metastases than do nondiabetic patients, whereas metformin users have rates of distant metastases similar to those of nondiabetic patients.

Treatment of HNC. Abbreviation: CRT: Chemoradiotherapy; DSS: Disease-specific survival; HNC: Head and neck cancer; Met+: Patients treated with metformin; Met−: Patients not treated with metformin; OPSCC: Oropharyngeal squamous cell carcinoma; OS: Overall survival; RAD: radiotherapy RFS: Recurrence-free survival; SCC: squamous cell carcinoma DSS: Disease-specific survival; RFS: recurrence-free survival.

## Data Availability

Data available on request.
